# Type 2 Diabetes and Fracture Risk in Older Women

**DOI:** 10.1001/jamanetworkopen.2024.25106

**Published:** 2024-08-06

**Authors:** Michail Zoulakis, Lisa Johansson, Henrik Litsne, Kristian Axelsson, Mattias Lorentzon

**Affiliations:** 1 Sahlgrenska Osteoporosis Centre, Institute of Medicine, University of Gothenburg, Gothenburg, Sweden; 2 Geriatric Medicine, Sahlgrenska University Hospital, Mölndal, Sweden; 3 Department of Orthopedics, Sahlgrenska University Hospital, Mölndal, Sweden; 4 Region Västra Götaland, Närhälsan Norrmalm, Health Centre, Sweden; 5 Mary MacKillop Institute for Health Research, Australian Catholic University, Fitzroy, Victoria, Australia

## Abstract

**Importance:**

The reasons for the increased fracture risk in type 2 diabetes (T2D) are not fully understood.

**Objective:**

To determine if poorer skeletal characteristics or worse physical function explain the increased fracture risk in T2D.

**Design, Setting, and Participants:**

This prospective observational study is based on the population-based Sahlgrenska University Hospital Prospective Evaluation of Risk of Bone Fractures study cohort of older women, performed in the Gothenburg area between March 2013 and May 2016. Follow-up of incident fracture data was completed in March 2023. Data analysis was performed between June and December 2023.

**Exposures:**

Data were collected from questionnaires and through examination of anthropometrics, physical function, and bone measurements using bone densitometry (dual-energy x-ray absorptiometry), and high-resolution peripheral computed tomography. A subsample underwent bone microindentation to assess bone material strength index (BMSi).

**Main Outcomes and Measures:**

Baseline assessment of bone characteristics and physical function and radiograph verified incident fractures.

**Results:**

Of 3008 women aged 75 to 80 years, 294 women with T2D (mean [SD] age, 77.8 [1.7] years) were compared with 2714 women without diabetes (mean [SD] age, 77.8 [1.6] years). Women with T2D had higher bone mineral density (BMD) at all sites (total hip, 4.4% higher; femoral neck (FN), 4.9% higher; and lumbar spine, 5.2% higher) than women without. At the tibia, women with T2D had 7.4% greater cortical area and 1.3% greater density, as well as 8.7% higher trabecular bone volume fraction. There was no difference in BMSi (T2D mean [SD], 78.0 [8.3] vs controls, 78.1 [7.3]). Women with T2D had lower performance on all physical function tests. The study found 9.7% lower grip strength, 9.9% slower gait speed, and 13.9% slower timed up-and-go time than women without diabetes. During a median (IQR) follow-up of 7.3 (4.4-8.4) years, 1071 incident fractures, 853 major osteoporotic fractures (MOF), and 232 hip fractures occurred. In adjusted (for age, body mass index, clinical risk factors, and FN BMD) Cox regression models, T2D was associated with an increased risk of any fracture (HR, 1.26; 95% CI, 1.04-1.54) and MOF (HR, 1.25; 95% CI, 1.00-1.56).

**Conclusions and Relevance:**

In this cohort study of older women, T2D was associated with higher BMD, better bone microarchitecture, and no different BMSi but poorer physical function, suggesting that poor physical function is the main reason for the increased fracture risk in T2D women.

## Key Points

**Question:**

Is poor physical function or an impaired skeletal phenotype associated with fracture risk in older women with type 2 diabetes (T2D)?

**Findings:**

In this cohort study, 3008 older women were included, of whom 294 had T2D. T2D was associated with increased fracture risk, higher bone mineral density, better bone microarchitecture, and similar bone material strength, but impaired physical function.

**Meaning:**

Results from this study suggest that the higher fracture risk among older women with T2D may be due to impaired physical function and not skeletal characteristics.

## Teaser

This cohort study evaluates whether poorer skeletal characteristics or worse physical function are associated with the increased fracture risk in women with type 2 diabetes.

## Introduction

Diabetes affects more than 500 million individuals worldwide,^1^ with recent studies indicating that the prevalence and burden will rise significantly in the future.^2^ Type 2 diabetes (T2D) made up 96.0% of all cases of diabetes globally.^1^ The impact of the disease is severe and includes progressive damage to organs, heart disease, kidney failure, stroke, atherosclerosis, peripheral neuropathy, visual impairment, and declining physical function.^3,4^

T2D prevalence has also been linked to increased fracture risk by several studies but to a different extent.^5,6,7^ A recent meta-analysis^7^ found an increased risk for fracture in both men and women with T2D. Two recent large cohort studies^5,6^ from Sweden demonstrated that the fracture risk was dependent on disease duration and diabetes medication.

The reasons for the increased fracture risk in patients with T2D are still not clear. Presented hypotheses suggest that T2D harms various bone properties that have a negative impact on fracture risk. Particularly, the accumulation of advanced glycation end products (AGEs) in bone has been proposed to be responsible for bone fragility in diabetes.^8,9,10^ Other potential underlying mechanisms have been proposed and include impaired bone turnover,^11,12,13^ different epigenetic regulators (MicroRNAs),^14,15^ increased levels of sclerostin^16,17^ or altered bone marrow adipose tissue.^18^

Paradoxically, patients with T2D have been shown to have higher bone mineral density (BMD)^19,20^ but worse cortical bone microstructure than individuals without diabetes.^21^ In particular, higher cortical porosity has been found in several publications^9,21,22,23,24,25,26,27^ including a study^28^ based on a subset of the Sahlgrenska University Hospital Prospective Evaluation of Risk of Bone Fractures (SUPERB) cohort.

It has been well established that impaired physical performance^28,29,30,31,32^ along with an increased propensity for falls^33,34,35^ are common in patients with T2D and could be important for the increased risk of fractures, although no prospective studies are available with comprehensive and detailed data on fracture risk, bone characteristics, and physical function. This study aims to determine if older women with T2D have increased fracture risk and if T2D is associated with impaired bone characteristics or with worse physical function.

## Methods

### Study Population

A total of 6382 women from the greater Gothenburg area aged 75 to 80 years were invited using information from the Swedish National Registry to participate in SUPERB, a prospective population-based study (eFigure in Supplement 1). Participants had to be able to walk, with or without an aid, understand Swedish, and have at least 1 hip that could be measured by dual-energy x-ray absorptiometry (DXA) to be eligible.

Of the 6832 women initially contacted, 3368 (52.6%) declined or did not respond, 436 had exclusion criteria, leaving 3028 (47.4%) women in the SUPERB cohort. Within this cohort, 20 individuals were found to have a confirmed diagnosis of type I diabetes and were consequently excluded from further analysis. Among the remaining participants, 294 individuals were identified as having known T2D (eMethods in Supplement 1). Data on all prescription medications were retrieved from the Swedish Prescribed Drug Register. The remaining 2714 women without diabetes composed the control group. The study adhered to the Strengthening the Reporting of Observational Studies in Epidemiology (STROBE) reporting guideline. The study was approved by the Gothenburg Regional ethics review board, and all participants provided verbal and written informed consent.

### Anthropometrics

Height and weight were measured using standardized equipment (eMethods in Supplement 1). Validated questionnaires were used to collect information on medical history, clinical risk factors (CRFs), previous fracture, smoking, parental history of hip fracture, oral glucocorticoid use, diabetes, rheumatoid arthritis, and alcohol use. Fracture Risk Assessment Tool (FRAX)–score calculations considered self-reported fractures that occurred after the age of 50 years at any site other than the skull. Medical history, including any previous or ongoing treatment, was also collected through questionnaires. In addition, the *International Statistical Classification of Diseases and Related Health Problems, Tenth Revision (ICD-10)* codes regarding prevalent diseases were collected using the National Patient Register, which collects diagnoses and procedures from inpatient and outpatient visits at Swedish hospitals. *ICD-10* codes and questionnaire data on prevalent diseases were used to calculate the Charlson comorbidity index to assess the comorbidity burden.^36^ Mortality data were obtained from the regional population registry Västfolket.

### Physical Activity and Physical Performance

The Physical Activity Scale for the Elderly (PASE)^37^ questionnaire was used to estimate physical activity over the previous 7 days before inclusion, and the 12-Item Short Form Health Survey^38^ was used to measure both physical health (PCS-12) and mental health.

Balance was assessed using the one leg standing (OLS) test (eMethods in Supplement 1),^39^ and functional mobility was measured using the timed up and go (TUG) test.^40^ The 30-second chair stand test was used to evaluate lower body strength and endurance.^41^ The 10-meter walk test was used to measure walking speed.^42^ Grip strength was measured as previously described.^28^

### Bone Densitometry and Vertebral Fracture Assessment

Areal BMD (aBMD) was measured using DXA as described in the eMethods in Supplement 1. Trabecular bone score (TBS) was calculated from the mean of the L1 to L4 vertebrae. Fractured vertebrae and those with osteosynthesis materials were excluded from the aBMD and TBS analyses. Vertebral fracture assessment (VFA) using lateral DXA images was used to detect vertebral fractures (eMethods in Supplement 1), which were then categorized and graded using the Genant semiquantitative system.^43^

### Assessment of Bone Quality and Biochemical Markers

Bone microarchitecture was measured using high-resolution peripheral quantitative computed tomography (HR-pQCT) imaging of the nondominant radius and ipsilateral tibia using the XtremeCT (Scanco Medical AG), as described in the eMethods in Supplement 1, and has been previously reported.^28,44^ Blood biochemistry analyses were performed at the clinical chemistry laboratory at the Sahlgrenska University Hospital (glycated hemoglobin [HbA_1c_]) and the Department of Clinical Chemistry (parathyroid hormone, 25-OH-vitamin D, and calcium) at the Linköping University Hospital, Sweden (eMethods in Supplement 1). Impact microindentation using the Osteoprobe (ActiveLife Scientific) device was performed at the mid tibia as previously described^44^ (eMethods in Supplement 1) in a subset of women to determine the bone material strength index (BMSi).

### Fractures, Mortality, and Medication

Information on incident fractures was retrieved from regional radiography archives for the Västra Götaland region and was evaluated from baseline (March 2013 to April 2016) through March 2023. To verify incident fractures, all radiology reports were examined. Incident fractures were divided into 3 categories: (1) major osteoporotic fractures (MOF, including hip, clinical spine, pelvis, wrist, and humerus fractures); (2) any fractures (except the skull, fingers, and toes); and (3) hip fractures. Mortality data were gathered from the Västfolket regional population register.

### Statistical Analysis

Differences between groups were investigated with independent samples *t* tests for continuous variables. For dichotomous variables, χ^2^ and Fisher exact test were used. Cox proportional hazard models investigated the association between groups (T2D vs control) and the incidence of fractures and death. Multivariable Cox models were adjusted for age, body mass index (BMI), CRFs (previous fragility fracture, parental hip fracture, smoking, alcohol consumption, glucocorticoids, rheumatoid arthritis, and secondary osteoporosis), previous osteoporosis medications (eMethods in Supplement 1), and femoral neck BMD. Hazard ratios (HR) and 95% CIs are presented. By visually reviewing the log (−log[survival]) vs log(time) curves for each outcome (any fracture, MOF, and hip fracture), the Cox models satisfied the proportionality assumption.

Sensitivity analyses were performed for subgroups defined by (1) T2D treatment type, (2) duration of diabetes treatment, (3) incident fracture status, (4) osteoporosis medication use, and (5) HbA_1c_ levels. Analysis of variance with Bonferroni post hoc tests to adjust for multiple comparisons were used to investigate differences between groups (eTable 3, eTable 4, eTable 5, and eTable 6 in Supplement 1).

Fine and Gray modeling was used to estimate associations with fracture risk while considering the competing risk of death. Statistical imputation using the multivariate imputation by chained equations package in RStudio was used for missing CRFs in FRAX using 20 iterations with Nelson-Aalen estimates for all the outcomes. Imputation was performed for 208 women (6.9%) with missing data on CRFs concerning 252 data points (eMethods in Supplement 1).

Two-sided *P* values less than .05 were considered significant. Statistical analyses were performed with SPSS version 29 (IBM) and RStudio version 1.4.1106 (Posit). Data were analyzed from June to December 2023.

## Results

### Baseline Characteristics

A total of 294 women with T2D (mean [SD] age, 77.8 [1.7] years) and 2714 women without diabetes (mean [SD] age, 77.8 [1.6] years) were included in this study. Women with T2D had 9.1% higher body weight, 9.5% higher BMI, and 6.3% higher appendicular lean mass index (lean mass divided by height squared) than controls. The T2D group had a lower prevalence of reported osteoporosis medication use compared with the controls (3.4% vs 7.5%, respectively). FRAX scores for the 10-year probabilities for MOF and hip fracture (with or without FN BMD) were consistently lower, while Charlson Comorbidity Index was higher in women with T2D than in those without (Table 1). A higher prevalence of stroke, ischemic heart disease, heart failure, and use of antihypertensive medication and statins was observed in the T2D group than in the control group (Table 1). Blood biochemistry analyses showed that 25-hydroxyvitamin D_3_ levels were 6.9% lower, while creatinine was 4.7% higher, calcium was 1.6% higher, and HbA_1c_ was 23.8% higher in the T2D group. There were no statistically significant differences between the T2D group and the control group in the prevalence of self-reported fractures or VFA-identified vertebral fractures (Table 1).

**Table 1.** Baseline Characteristics

Characteristic	Participants, No. (%)		*P* value
	T2D (n = 294)	Control (n = 2714)	
Age, mean (SD), y	77.8 (1.7)	77.8 (1.6)	.48
Weight, mean (SD), kg	74.6 (13.6)	68.1 (11.8)	<.001
Height, mean (SD), cm	161.6 (58.2)	161.8 (59.0)	.56
Body mass index, mean (SD)^a^	28.6 (4.9)	26.0 (4.3)	<.001
ALMi, mean (SD), kg/m^2^	6.6 (0.98)^b^	6.2 (0.83)^c^	<.001
Current smoking	16 (5.4)	140 (5.2)	.83
Excessive alcohol intake^d^	2 (0.7)	15 (0.6)	.78
Parental hip fracture	50 (17.0)	480 (17.7)	.77
Previous fractures	96 (32.7)	1014 (37.4)	.11
Vertebral fracture on VFA	69 (24.6)	633 (24.1)	.87
Oral glucocorticoid use	8 (2.7)	94 (3.5)	.50
Secondary osteoporosis^e^	56 (19)	486 (17.9)	.83
Previous osteoporosis treatment^f^	10 (3.4)^g^	202 (7.5)^h^	.01
Current osteoporosis treatment	27 (9.2)^g^	293 (10.8)^h^	.23
Rheumatoid arthritis	13 (4.4)	104 (3.8)	.62
Calcium intake, median (IQR), mg/d	345 (0-605)	393 (0-623)	.51
FRAX score, mean (SD)			
MOF with BMD	21.1 (11.3)	23.2 (11.9)	.003
MOF without BMD	30.6 (12.4)	33.0 (13.0)	.002
Hip with BMD	9.6 (10.3)	11.2 (11.1)	.01
Hip without BMD	17.7 (11.9)	20.0 (13.5)	.002
Charlson Comorbidity Index, mean (SD)	1.49 (1.85)	0.64 (1.28)	<.001
0	111 (37.8)	1875 (69.1)	<.001
1	66 (22.4)	288 (10.6)	<.001
2	54 (18.4)	415 (15.3)	.17
3	37 (12.6)	73 (2.7)	<.001
≥4	26 (8.8)	63 (2.3)	<.001
Medication			
Antihypertensive treatment	232 (78.9)	1288 (47.5)	<.001
Statins	238 (81)	1107 (40.8)	<.001
Medical history			
Ischemic heart disease^i^	29 (9.9)	157 (5.8)	.01
Heart failure^j^	18 (6.1)	48 (1.8)	<.001
Cardiovascular disease^k^	13 (4.4)	95 (3.3)	.25
Blood biochemistry			
25-Hydroxyvitamin D_3_, ng/ml	23.48 (8.13)	25.16 (8.53)	.001
Parathyroid hormone, pg/ml	49.04 (25.46)	47.15 (19.80)	.33
Creatinine, mg/dL	0.91 (0.26)	0.87 (0.20)	.01
Calcium, mg/dL	10.04 (0.44)	9.88 (0.40)	<.001
HbA_1c_, median (IQR), mmol/mol	47 (42-53)^l^	37 (35-37)^m^	<.001

Abbreviations: ALMi, appendicular lean mass/height squared; BMD, bone mineral density; FRAX, Fracture Risk Assessment Tool; HbA_1c_, glycated hemoglobin; MOF, major osteoporotic fracture; T2D, type 2 diabetes; VFA, Vertebral Fracture Assessment.

SI conversion factors: To convert 25-Hydroxyvitamin D_3_ to nanomoles per liter, mutiply by 2.496; calcium to millimoles per liter, multiply by 0.25; creatinine to micromoles per liter, multiply by 88.4; parathyroid hormone to nanograms per liter, multiply by 1.

^a^ Body mass index is calculated as weight in kilograms divided by height in meters squared.

^b^ Data available for 292 participants.

^c^ Data available for 2704 participants.

^d^ Twenty-one or more units per week.

^e^ Includes hyperthyroidism, malnutrition, osteogenesis imperfecta, chronic liver disease, premature menopause, and hyperparathyroidism.

^f^ Previous treatment with bisphosphonates, teriparatide, or denosumab.

^g^ Data available for 293 participants.

^h^ Data available for 2710 participants.

^i^ *International Statistical Classification of Diseases and Related Health Problems, Tenth Revision* (*ICD-10*) codes I20 to I25.

^j^ *ICD-10* code I50.

^k^ *ICD-10* codes I60 to I69.

^l^ Data available for 103 participants.

^m^ Data available for 896 participants.

### Bone Characteristics Measured by DXA and HR-pQCT

Women with T2D had higher BMD in the total hip (4.4% higher), femoral neck (4.9% higher) and lumbar spine (5.2% higher) compared with controls, although TBS was 1.6% lower. There was no difference in BMSi between groups. The associations for BMD of the total hip and lumbar spine remained after adjustment for age and BMI (Table 2).

**Table 2.** Bone Characteristics and Physical Function Tests

Characteristic	Mean (SD)		Difference, %	*P* value	Adjusted unstandardized β (95% CI)^a^	*P* value
	T2D (n = 293)	Control (n = 2702)				
BMD femoral neck, g/cm^2^	0.69 (0.12)	0.66 (0.10)	4.4	<.001	0.010 (−0.002 to 0.023)	.10
BMD hip total, g/cm^2^	0.84 (0.13)	0.80 (0.11)	4.9	<.001	0.017 (0.004 to 0.030)	.01
BMD lumbar spine, g/cm^2^	0.99 (0.18)^b^	0.94 (0.17)^c^	5.2	<.001	0.026 (0.006 to 0.046)	.01
BMSi	78.0 (8.3)^d^	78.1 (7.3)^e^	0.1	.93	0.93 (−1.053 to 2.912)	.36
Trabecular bone score	1.19 (0.1)^b^	1.21 (0.1)^c^	1.6	.01	−0.004 (−0.017 to 0.009)	.53
HR-pQCT ultradistal tibia^f^						
Total vBMD, mg/cm^3^	243.8 (52.4)	224.1 (47.2)	8.4	<.001	13.36 (7.63 to 19.09)	<.001
Cortical area, mm^2^	83.6 (24.5)	77.6 (23.1)^g^	7.4	<.001	2.42 (−0.33 to 5.18	.08
Cortical vBMD, mg/cm^3^	747.7 (68.8)	738.3 (68.8)	1.3	.03	3.44 (−4.97 to 11.86)	.42
Cortical porosity, %	12.3 (4.0)	12.2 (3.9)^h^	0.8	.39	0.2 (−0.3 to 0.6)	.53
Trabecular BV/TV, %	13.2 (3.0)	12.1 (2.9)	8.7	<.001	0.82 (0.46 to 1.17)	<.001
Trabecular thickness, mm	0.071 (0.01)	0.069 (0.02)	2.9	.003	0.003 (0.002 to 0.005)	<.001
Trabecular separation, mm	0.48 (0.13)	0.52 (0.13)^i^	6.8	<.001	−0.010 (−0.025 to 0.006)	.22
Stiffness, kN/mm	172 (30)	162 (29)^j^	5.5	<.001	4.51 (1.11 to 7.92)	.01
Failure load, kN	8713 (1453	8272 (1396)^i^	5.2	<.001	196.8 (32.4 to 361.1)	.02
Physical function^k^						
PCS-12	42 (11.1)	45.5 (10.8)	−8.0	<.001	−1.96 (−3.24 to −0.68)	.003
MCS-12, median (IQR)	55.9 (46.8 to 60.6)	56.1 (49.1 to 59.8)	−0.4	.46	−1.12 (−2.27 to 0.02)	.01
PASE, median (IQR)	81.0 (55.0 to 121.4)	98.2 (67.6 to 136.0)^l^	−19.2	<.001	−8.49 (−14.54 to −2.45)	.01
Previous falls, No. (%)	95 (32.4)	792 (29.2)^m^	10.4	.25	0.02 (−0.03 to 0.08)	.44
Timed up go, s	9.1 (3.9)^n^	7.9 (2.5)^o^	13.9	<.001	0.78 (0.40 to 1.17)	<.001
Walking speed, m/s	1.15 (0.26)^p^	1.27 (0.25)^o^	−9.9	<.001	−0.07 (−0.10 to −0.04)	<.001
Grip strength, kg	13.5 (5.3)^q^	14.9 (5.5)^r^	−9.7	<.001	−1.21 (−1.89 to −0.53)	<.001
One leg standing, s	10.8 (8.2)^s^	14.2 (9.7)^t^	−27.2	<.001	−2.21 (−3.56 to −0.86)	.001
30-s Chair stand, n	9.0 (4.5)^u^	10.7 (4.3)^v^	−17.3	<.001	−1.08 (−1.60 to −0.57)	<.001

Abbreviations: BMD, bone mineral density; BMSi, bone material strength index; BV/TV, trabecular bone volume to total volume fraction; HR-pQCT, high-resolution peripheral quantitative computed tomography; MCS-12, Mental Component Score; PASE, Physical Activity Scale for the Elderly; PCS-12, Physical Component Score; T2D, type 2 diabetes; vBMD, volumetric BMD.

^a^ Associations between T2D and outcome variables are presented as unstandardized β derived from linear regression models adjusted for age and body mass index, with bone characteristics or physical function tests as dependent variables.

^b^ Data available for 290 participants.

^c^ Data available for 2692 participants.

^d^ Data available for 57 participants.

^e^ Data available for 587 participants.

^f^ The denominator for HR-pQCT ultradistal tibia is 283 for T2D and 2626 for the control group.

^g^ Data available for 2627 participants.

^h^ Data available for 2623 participants.

^i^ Data available for 2615 participants.

^j^ Data available for 2625 participants.

^k^ The denominator for physical function is 293 for T2D and 2707 for the control group.

^l^ Data available for 2701 participants.

^m^ Data available for 2712 participants.

^n^ Data available for 291 participants.

^o^ Data available for 2695 participants.

^p^ Data available for 292 participants.

^q^ Data available for 277 participants.

^r^ Data available for 2623 participants.

^s^ Data available for 189 participants.

^t^ Data available for 2210 participants.

^u^ Data available for 289 participants.

^v^ Data available for 2688 participants.

At the ultradistal site, the T2D group had a 7.4% higher cortical area, 8.4% higher total vBMD, 1.3% higher cortical volumetric BMD (ct.vBMD), 8.7% higher trabecular bone volume fraction (BV/TV), 2.9% higher trabecular thickness, and 6.8% lower trabecular separation compared with patients without diabetes. Greater stiffness (3.8% to 8.8%, depending on site) and higher ultimate failure load (3.9% to 8.1%) were observed at all sites in the T2D group compared with the control group (Table 2). The associations for bone strength indices, total vBMD, and trabecular BV/TV remained after adjustments for age and BMI. Similar associations between T2D and bone characteristics were found when analyzing the ultradistal radius, distal radius, and distal tibia (eTable 1 in Supplement 1).

### Physical Function

All physical function metrics and physical activity levels were significantly worse in the T2D group than in the control group. Patients with T2D had lower PCS-12 and PASE scores (8.0% and 19.2% lower, respectively). Women with T2D had 27.2% shorter OLS, 13.9% longer TUG, 9.9% slower walking speed, 17.3% fewer rises on the 30-second chair stand test, and 9.7% lower grip strength (Table 2). All associations observed for the physical function characteristics remained after adjustments for age and BMI.

### Association Between T2D and Incident Fractures

During a median (IQR) 7.3 (4.4-8.4) years of follow-up, 1071 incident any fractures, 853 MOFs, and 232 hip fractures occurred (eTable 2 in Supplement 1). In fully adjusted Cox regression models, T2D was associated with an increased risk of any fracture (HR, 1.26; 95% CI, 1.04-1.54) and MOF (HR, 1.25; 95% CI, 1.00-1.56), while the risk for hip fracture did not reach statistical significance (HR, 1.31; 95% CI, 0.86-2.01).

### Analysis per T2D Medication and Duration

**Figure 1.**
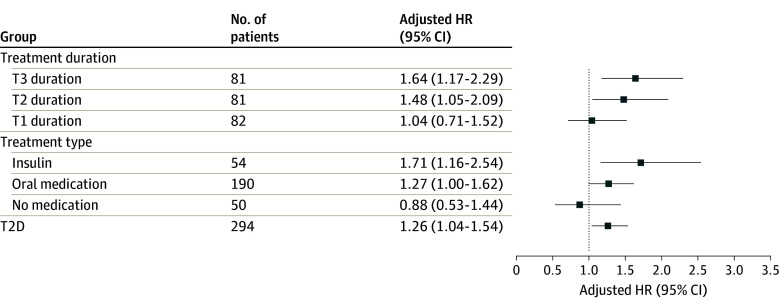
The Risk of Any Fracture in Type 2 Diabetes (T2D) According to Diabetes Duration and Treatment The relative risk of any fracture in T2D subgroups, according to diabetes duration and treatment, compared with women without diabetes using adjusted Cox proportional hazards regression models. HR indicates hazard ratio; T, tertile.

**Figure 2.**
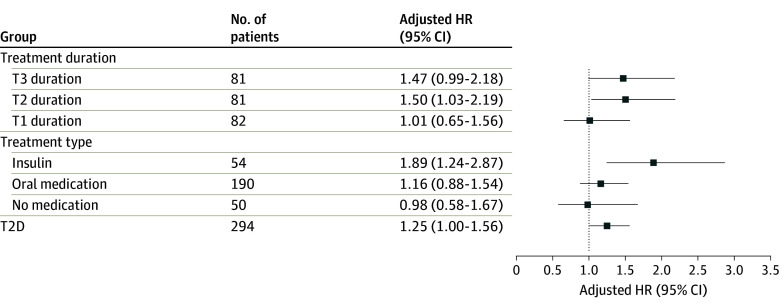
The Risk of Major Osteoporotic Fracture in Type 2 Diabetes (T2D) According to Diabetes Duration and Treatment The relative risk of major osteoporotic fracture (MOF) in T2D subgroups, according to diabetes duration and treatment, compared with women without diabetes using adjusted Cox proportional hazards regression models. HR indicates hazard ratio; T, tertile.

**Figure 3.**
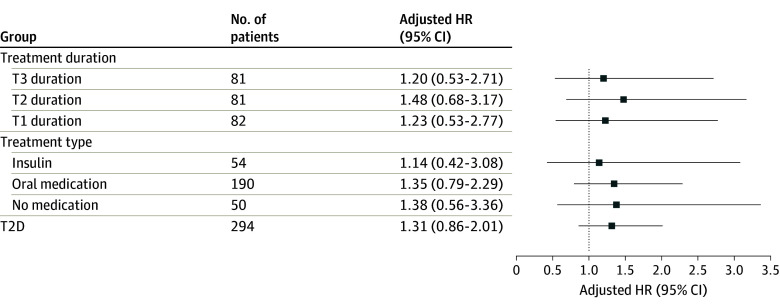
The Risk of Hip Fracture in Type 2 Diabetes (T2D) According to Diabetes Duration and Treatment The relative risk of hip fracture in T2D subgroups, according to diabetes duration and treatment, compared with women without diabetes using adjusted Cox proportional hazards regression models. HR indicates hazard ratio; T, tertile.

Women taking T2D medication had higher aBMD, greater bone strength, and better bone microarchitecture, but poorer physical performance than controls (eTable 3 in Supplement 1). Fully adjusted Cox regression models found an increased fracture risk in women treated with insulin, compared with controls, for any fracture (HR, 1.71; 95% CI, 1.16-2.54) and MOF (HR, 1.89; 95% CI, 1.24-2.87) but not for hip fracture (HR, 1.14; 95% CI, 0.42-3.08). Women with oral T2D treatment had a higher risk of any fracture (HR, 1.27; 95% CI, 1.00-1.62) but not of MOF (1.16; 95% CI, 0.88-1.54) or hip fracture (HR 1.35; 95% CI, 0.79-2.29) (Figure 1, Figure 2, and Figure 3).

Women who had T2D medication were further divided into tertiles, representing the length of their treatment duration (eTable 4 in Supplement 1). Areal BMD was higher in all tertiles of patients with T2D duration compared with controls without diabetes, but there were no differences between the T2D duration groups. No significant differences were observed in bone microarchitecture between the different T2D duration groups (eTable 5 in Supplement 1).

Most indices reflecting physical function were worse in T2D duration groups than in the control group (eTable 4 in Supplement 1). A fully adjusted Cox regression model revealed that women in tertile 2 and tertile 3 had the highest risk of any fracture (HR, 1.48; 95% CI, 1.05-2.09 and HR, 1.64; 95% CI, 1.17-2.29, respectively) and MOF (HR, 1.50; 95% CI, 1.03-2.19 and HR 1.47; 95% CI, 0.99-2.18, respectively) (Figure 1, Figure 2, and Figure 3).

### Women With and Without Incident Fractures

Four groups were examined in a sensitivity analysis according to their T2D status and incident fracture during follow-up (eTable 5 in Supplement 1). Women with T2D and incident fractures had significantly greater total hip and lumbar spine aBMD, higher ultradistal failure load and stiffness, as well as significantly worse TUG, chair stand test, walking speed, grip strength, and OLS than controls with incident fractures. Similar and significant differences were observed between women with T2D without incident fractures, except grip strength, compared with controls without incident fracture (eTable 5 in Supplement 1). A higher frequency of self-reported falls in the last year was observed in women with T2D and incident fracture than in T2D women without fracture and in controls without fracture (eTable 6 in Supplement 1).

### Additional Analyses

Cox regression analyses excluding women with ongoing osteoporosis medication at baseline (eTable 7 in Supplement 1) or previous osteoporosis treatment (eTable 8 in Supplement 1) revealed that T2D was associated with an increased risk of any fracture in fully adjusted models. In a subset of 103 women with T2D and 896 controls with available HbA_1c_, Cox regression models demonstrated that women with T2D in the third tertile of HbA_1c_ had a significantly higher risk of any fracture (HR, 2.34; 95% CI, 1.35-4.07) and hip fracture (HR, 4.56; 95% CI, 1.59-13.03) than the controls (eTable 9 in Supplement 1).

### T2D and Mortality

T2D was associated with increased mortality risk (HR for death, 1.54; 95% CI, 1.21-1.97) (eTable 10 in Supplement 1). A higher risk of death was also observed in the third tertile of T2D duration (HR, 1.75; 95% CI, 1.12-2.71) and in those taking diabetes medication (insulin HR, 2.01; 95% CI, 1.25-3.23; and oral medications HR, 1.40; 95 CI, 1.03-1.91).

The competing risk of death was accounted for using a multivariate-adjusted Fine and Grey analysis. The subdistribution HR (SHR) for MOF was 1.21 (95% CI, 0.98-1.50), the SHR for any fracture was 1.21 (95% CI, 1.00-1.47), and the SHR for hip fracture was 1.25 (95% CI, 0.82-1.91) (eTable 11 in Supplement 1).

## Discussion

Older women with T2D had higher BMD, better bone microarchitecture, and nondifferent BMSi, but poorer physical performance and higher fracture risk than women without diabetes. Subgroup analyses revealed that prolonged diabetes treatment and insulin use were associated with higher fracture risk and worse physical performance while bone characteristics were better.

Our findings regarding BMD are consistent with previous publications showing higher BMD in individuals with T2D compared with those without diabetes.^19,20^ Notably, patients receiving insulin or oral treatment exhibited the highest BMD, while treatment duration did not show a significant association with BMD. The observed higher BMD values were independent of body weight but may be attributed to factors such as obesity, hyperinsulinemia, and altered adipokine levels.^11^ This observation suggests that BMD per se cannot explain the increased fracture risk in T2D.

In contrast, TBS was slightly lower in T2D compared with controls (1.6%), as reported previously,^19,45^ suggesting that some abnormalities in the trabecular bone structure in T2D may exist, although this difference may also be due to high BMI-related errors in the TBS measurements.^46^ The latter is supported by the obtained HR-pQCT results, showing higher trabecular bone volume fraction in women with T2D, in agreement with previous reports.^9,23,24,25^ A recent meta-analysis^21^ reported similar findings and suggests that the improved trabecular structures could be a compensatory mechanism for cortical weakness, a hypothesis refuted by the results obtained in our cohort, demonstrating that cortical parameters such as cortical area and density were also better in women with T2D.

In a smaller subset of this cohort, higher cortical porosity and lower BMSi in women with T2D were previously reported.^28^ Yet, a full cohort analysis with more power calls these findings into question. Additionally, no disparities in BMSi were observed in the subgroup analysis by T2D medication and duration. Other studies with smaller sample sizes have reported associations between BMSi and T2D.^8,47^ In line with our findings, Khosla et al^9^ initially reported lower BMSi in patients with T2D, but a larger sample size negated this difference. The original association between BMSi and T2D may have been influenced by insufficient statistical power. Population-based cohorts like SUPERB, which include all participants under consistent criteria, may provide more reliable associations than smaller case-control studies.

As previously demonstrated,^48^ T2D was associated with an increased mortality risk, which could affect the results of the study. However, both Cox regression models and Fine and Grey models, considering the competing risk of death, found similarly increased risk of fracture in women with T2D. Lower vitamin D levels were observed in women with T2D, consistent with previous findings, and may be associated with a higher prevalence of obesity and lower physical activity levels in women with T2D compared with controls.^49,50^

Previous studies^51,52,53^ have indicated that inadequate glycemic control is linked to an elevated risk of fractures in individuals with T2D. This finding was supported by a subgroup analysis of HbA_1c_ levels in the present study, which found that women in the third tertile of HbA_1c_ levels had an elevated risk of any fracture and hip fracture.

In this study, Cox regression analyses demonstrated that T2D was associated with a higher risk of any fracture and major osteoporotic fracture, but not with hip fracture, probably due to insufficient power for the latter. Sensitivity analyses found that individuals receiving insulin treatment and those with a longer duration of T2D exhibited a higher HR for fractures. Conversely, women with T2D who were not receiving medical treatment or had a shorter treatment duration showed no elevated risk for fractures, a finding supported by recent large cohort studies from Sweden.^5,6^ Results from this study agree with findings from a comprehensive analysis^6^ of 580 127 Swedish patients with T2D, reporting that insulin treatment and a long duration of T2D were associated with increased fracture risk. To assess the reliability of our findings, an additional sensitivity analysis for groups with and without incident fractures demonstrated that women with T2D with fractures had better or equal bone parameters compared with controls with incident fractures.

Previous findings from the SUPERB cohort have demonstrated that physical performance tests including TUG and OLS are independently associated with fracture risk in the SUPERB cohort.^31,32^ Physical activity has been previously associated with falls and fracture risk in older women,^54,55^ and T2D has been associated with low physical activity and an increased risk of falling.^28,33,34,35^ Thus, the results from the present study and previous evidence demonstrate that physical activity is lower, and physical performance is impaired in T2D, and it is clear that poor physical performance is independently associated with fracture risk. It was therefore hypothesized that reduced physical performance, and not impaired bone health, is the underlying reason for the increased risk of fracture observed in T2D. In support of this hypothesis, our observations revealed a decline in performance on physical function tests and indicators of physical activity (PASE) and physical health (PCS-12), but no impairments in detailed bone characteristics.

### Limitations and Strengths

This study had limitations. The population studied was composed of older women, and the findings may not generalize to other groups. Second, this was an observational study, and causal relationships cannot be inferred. Another limitation is the lack of measurements of AGEs accumulation, which has been linked to adverse effects on bone material properties as well as diabetic neuropathy and myopathy,^8,9,10,11,56^ resulting in physical deterioration and postural instability, all of which increase the risk of falls and loss of mobility. The lack of differences in BMSi between women with and without T2D argues against an AGEs-mediated negative effect on bone material strength in women with T2D. The number of comparisons performed on the cross-sectional baseline data was large (Table 2) but most associations regarding bone characteristics and physical function tests would remain, also after adjustment for all baseline comparisons made, supporting that the observed associations were not due to chance alone.

Finally, the reliability of self-reported previous falls is low and the prevalence of reported falls may not accurately identify individuals at risk.^57^ In contrast, radiographically confirmed fractures offer a more reliable indicator of fall risk due to their objective verification, which could be the reason that fracture risk and not fall history was significantly higher in the T2D group than in the controls.

This study also has strengths. It is the largest population-based study investigated that we know of with detailed data on bone characteristics, including detailed bone characteristics, comorbidities, physical function, medications, and fractures. Availability of radiograph-verified fractures with high accuracy as well as a minimal loss to follow-up by using national registers and a radiograph archive increases the reliability of the results.

## Conclusions

Older women with T2D have higher BMD, better bone microarchitecture, and no different BMSi, but poorer physical function than women without diabetes. This could be the principal reason for the increased fracture risk observed in this study among older women with T2D.

## Notes

Supplement 1. **eMethods. **Additional Information on Study Methods **eFigure. **Study Population **eTable 1. **Bone Microarchitecture in Older Women With T2D and Controls **eTable 2. **Fracture Outcomes for Women With T2D vs Controls **eTable 3. **Characteristics According to T2D Medication Type **eTable 4. **Characteristics According to T2D Duration **eTable 5. **Characteristics According to Incident Fracture **eTable 6. **Baseline Characteristics According to Incident Fracture **eTable 7. **Fracture Outcomes for Women With T2D vs Controls Without Osteoporosis Treatment at Baseline **eTable 8. **Fracture Outcomes for Women With T2D vs Controls Without Previous Osteoporosis Treatment **eTable 9. **Fractures and Mortality per Tertile of HbA1c **eTable 10. **Mortality and T2D **eTable 11. **Fine and Gray Subdistribution Hazard Ratios for T2D Women vs Controls
eReferences.

Supplement 2.
Data Sharing Statement
