# Supplementary material for: Type 2 Diabetes and Fracture Risk in Older Women
Source: JAMA Netw Open. 2024 Aug 6;7(8):e2425106. doi: 10.1001/jamanetworkopen.2024.25106 (PMC11304123; doi:10.1001/jamanetworkopen.2024.25106)
Supplement: Supplement 1. — eMethods. Additional Information on Study Methods eFigure. Study Population eTable 1. Bone Microarchitecture in Older Women With T2D and Controls eTable 2. Fracture Outcomes for Women With T2D vs Controls eTable 3. Characteristics According to T2D Medication Type eTable 4. Characteristics According to T2D Duration eTable 5. Characteristics According to Incident Fracture eTable 6. Baseline Characteristics According to Incident Fracture eTable 7. Fracture Outcomes for Women With T2D vs Controls Without Osteoporosis Treatment at Baseline eTable 8. Fracture Outcomes for Women With T2D vs Controls Without Previous Osteoporosis Treatment eTable 9. Fractures and Mortality per Tertile of HbA1c eTable 10. Mortality and T2D eTable 11. Fine and Gray Subdistribution Hazard Ratios for T2D Women vs Controls eReferences. [file jamanetwopen-e2425106-s001.pdf]

Supplemental Online Content

Zoulakis M, Johansson L, Litsne H, Axelsson K, Lorentzon M. Type 2 diabetes and fracture risk in older women. *JAMA Netw Open*. 2024;7(8):e2425106.
doi:10.1001/jamanetworkopen.2024.25106

eMethods. Additional Information on Study Methods
eFigure. Study Population
eTable 1. Bone Microarchitecture in Older Women with T2D and Controls
eTable 2. Fracture Outcomes for Women with T2D vs Controls
eTable 3. Characteristics According to T2D Medication Type
eTable 4. Characteristics According to T2D Duration
eTable 5. Characteristics According to Incident Fracture
eTable 6. Baseline Characteristics According to Incident Fracture
eTable 7. Fracture Outcomes for Women With T2D vs Controls Without Osteoporosis Treatment at Baseline
eTable 8. Fracture Outcomes for Women With T2D vs. Controls Without Previous Osteoporosis Treatment
eTable 9. Fractures and Mortality per Tertile of HbA1c
eTable 10. Mortality and T2D
eTable 11. Fine and Gray Subdistribution Hazard Ratios for T2D Women vs Controls
eReferences.

This supplemental material has been provided by the authors to give readers additional information about their work.

© 2024 Zoulakis M et al. *JAMA Network Open.*

eMethods. Additional Information on Study Methods

Definition of the diabetes population

All women who had a self-reported diagnosis or confirmed treatment of diabetes mellitus (totaling 296 individuals) were considered for the Type 2 Diabetes Mellitus (T2D) group. The National Patient Register was used for validation purposes, utilizing ICD-10 codes to review the medical history of T2D individuals to determine the duration, up to five years prior to the baseline exam. Data from this analysis identified 20 women diagnosed with E10 (type 1 diabetes) who were excluded from further analyses. A confirmed diagnosis of E11 (T2D) was established for 264 women. An additional 15 women without self-reported diagnosis had an E11 diagnosis and were added to the T2D group. For additional validation and sensitivity analysis, the National Drug Register was utilized. The analysis revealed that 54 women were prescribed insulin therapy, either as monotherapy or as part of a polytherapy regimen. Additionally, 190 women were found to be exclusively on oral diabetes medication. Of the 15 women included in this study by ICD diagnosis, 12 were confirmed to be receiving diabetes medication. Furthermore, 3 women who had at least 2 prescriptions for T2D treatment in the 6 months prior to their inclusion were added to the T2D group.

Anthropometrics

Two measurements of body height were taken using a wall-mounted stadiometer. For all women, body weight was calculated to the nearest 0.1 kg using the same standard scale.

Physical Activity and Performance Measurements

© 2024 Zoulakis M et al. *JAMA Network Open.*

The Physical Activity Scale for the Elderly (PASE)[1] was utilized to estimate physical activity over the previous 7 days before inclusion, with a higher PASE score indicating a higher level of physical activity. In addition, the SF-12 Health survey[2] was used to measure both physical health (PCS-12) and mental health (MCS-12).

Balance was assessed using the One Leg Standing (OLS) test,[3–6] which measured the maximum time of balancing on one leg with arms crossed against the chest. After allowing for a practice round, the test was performed twice for each leg. The average time using the best time of each leg was then calculated. 609 either declined or were unable to perform this test.

The Timed Up and Go (TUG) test [7–9] was used to measure functional mobility and balance, by measuring the time, a participant needed to rise from an armchair, walk a three-meter distance, return, and sit down in the same chair. 14 participants were unable to perform the test.

The 30-s chair stand test[10] was used to evaluate lower body strength and endurance. The total number of stands a participant could perform from a chair within 30 seconds while having their arms crossed over the chest was measured. The 10-meter walk test was used to measure walking speed, and reflects functional mobility, gait, and vestibular function. [11,12,13] The participants walked without assistance for 10 meters, with the time measured for the intermediate 6 meters, allowing for 2 seconds of acceleration and 2 seconds of deceleration. The test was performed twice, and the average was used to calculate the walking speed. 49 participants declined or failed to perform these tests.

A Saehan hydraulic hand dynamometer (model SH5001; Saehan Corporation, Masan, South Korea), was used to measure arm grip strength, as a proxy for upper body muscular strength.[14]

© 2024 Zoulakis M et al. *JAMA Network Open.*

With the lower arm lying on a flat surface and the elbow held at a 90° angle, each participant completed two tries. Either the left or the right hand's maximum value of the two means was used.108 participants failed to perform this test.

Bone densitometry

2995 women had their areal BMD (aBMD) measured using the same dual-energy x-ray absorptiometry (DXA) device (Hologic discovery, Hologic, Waltham, MA), and 33 women were measured on a Hologic QDR 4500/A Delphi DXA device. Cross-calibration between the two machines was carried out, as previously described.[15] For all analyses, aBMD (g/cm2) of the lumbar spine L1-L4 (LS) and femoral neck (FN) were used.

Vertebral Fracture Assessment (VFA)

Vertebral fracture assessment using lateral DXA images was used to detect vertebral fractures, which were then categorized and graded using Genant's semi-quantitative system.[16] Two qualified medical professionals assessed the vertebral fractures as previously described.[17,18] The vertebral fracture diagnosis using VFA had an intra-observer agreement of 98.9%, and for moderate to severe vertebral fractures, the agreement was 100%.[17,18]

High-Resolution peripheral Quantitative Computed Tomography (HR-pQCT)

In cases where the ipsilateral tibia was fractured, the contralateral side was chosen instead. Measurements were obtained from the standard ultradistal site (9.5 mm and 22.5 mm from the

© 2024 Zoulakis M et al. *JAMA Network Open.*

reference line for the radius and tibia, respectively) and at the distal site (14% of measured bone length, measured from the end plate) of each bone.

At each site, 110 parallel slices, with a nominal isotropic resolution of 82 μm, were obtained which enabled the construction of the 3D bone model. Every stack of images was graded from 1-5 representing the level of image quality. Images with the highest quality were graded 1 and those with the lowest quality were graded 5. As per standard protocol, only images of adequate quality (grades 1-3) were used for further analyses. Image procession generated the following variables: total volumetric BMD (TtBMD, mg/cm^3), cortical area (Ct.Ar, mm^2), cortical thickness (Ct.Th, mm), cortical volumetric bone mineral density (Ct.BMD, mg/cm^3), trabecular bone volume fraction (BV/TV, %), trabecular thickness (Tb.Th, mm), and separation (Tb.Sp, mm).

An Image Processing Language (IPL v5.08b), provided by the manufacturer was utilized for further analysis to obtain variables needed to calculate cortical porosity (Ct.po) which was then calculated using the following equation: cortical pore volume/(cortical pore volume + cortical bone volume. As we have explained in previous publications,[19,20] it was necessary to separate bone tissue and cortical bone from extraosseous tissue and trabecular bone by placing contours in the endosteal and periosteal surface of the cortical bone. Calculations of stiffness (the resistance against deformation) and ultimate failure load were obtained with the use of the manufacturer's finite element analysis with the conversion of voxels to equally sized brick elements. Failure load was defined as the load at which at least 2% of the bone elements exceeded, 7000 microstrains during simulated uniaxial compression. Stiffness, the resistance against deformation, was analyzed. A Young's modulus of 10 GPa and a Poisson ratio of 0.3 were used in the FE models for all participants, as previously described.[21] The CVs for measurement of

trabecular parameters in the distal radius and distal tibia were 0.4% to 2.5% and 0.8% to 2.6%, respectively. CVs for measurement of cortical parameters in the distal radius and distal tibia were 0.1% to 0.9%.

Serum Analyses

Vitamin D 25-OH-D (nmol /l^{-1}) levels were measured from serum samples using chemiluminescence immunoassay (DiaSorin, Stillwater, MN, USA), and total CVs of 8.8%, 6.4%, and 6.8% at levels 25 nmol/L, 68 nmol/L, and 150 nmol/L, respectively. PTH was analyzed with Elecsys electrochemiluminescence immunoassay using a Roche Cobas e601 (Roche Diagnostics Scandinavia AB, Gothenburg, Sweden), CV of 4.0%, and 2.9% at levels 1.9 pmol/L and 8.6 pmol/L, respectively. Serum creatinine was measured using CREP2 on a Cobas 6000 instrument, with a CV of 4% at concentrations 85 and 400 μM. HbA1c was measured using HPLC (Mono S, Tricorn 50/50 GL (CDP), MonoBeads Column (GE Healthcare)). The separated haemoglobin fractions were measured using a UV-detector and absorbance was quantified at 417 nm. The CV was 2% at concentrations 42 mmol per mol, 63 mmol per mol and 94 mmol per mol.

Bone Microindentation

The Osteoprobe device was not available at the study start but became available later on with measurements of the first women starting in September 2014. From the initial 3028 women, only the latter 1613 were asked to participate and 647 (40% inclusion rate) were accepted to undergo bone micro-indentation. This procedure has been described in more detail previously.[20] Indentations were performed after application of a local anesthetic, at least 11 indentations were

© 2024 Zoulakis M et al. *JAMA Network Open.*

performed at the mid-shaft of the tibia in a circular manner and with a minimum 2 mm distance. To ensure that the probe was completely established on the cortical surface the first indentation was discarded, the remaining indentations were visually attested and indentations deviating due to technical or mechanical reasons were removed. Five operators in total carried out the procedure and during the first 100 measurements, at least two operators were present to ensure that everyone conducted the process consistently. The intra-observer CV (using the same operator at a different site) was 3.2%, while the inter-observer CV (using different operators) was 5.2%.

Statistical Analyses

Previous osteoporosis medication was defined as previous treatment with oral bisphosphonates, zoledronic acid, denosumab, strontium ranelate, and parathyroid hormone analogs.

Imputation was conducted for a total of 252 missing data points regarding the risk factors in the Fracture Risk Assessment Tool FRAX (49 regarding missing information on parental history of hip fracture, 7 for rheumatoid arthritis, 9 for previous fragility fracture, 4 for current smoking, 3 for excessive alcohol usage,7 for oral glucocorticoids and 176 for secondary osteoporosis (147 for premature menopause, 11 for inflammatory bowel disease, 6 for hyperthyroidism and 9 for chronic liver disease).

© 2024 Zoulakis M et al. *JAMA Network Open.*

eFigure Study population

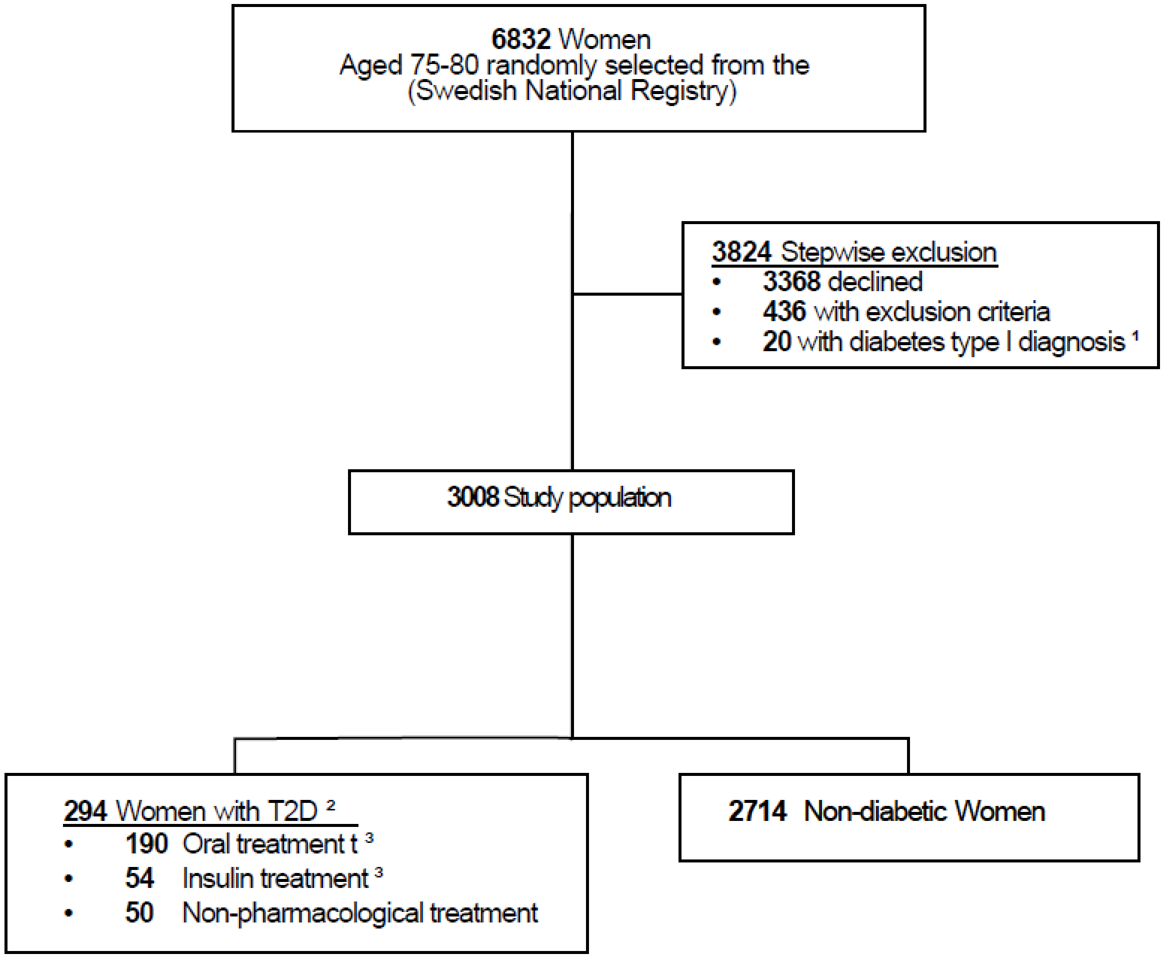

1 International classification code (ICD-10),E10.-

2 276 self-reported T2D diagnosis,
15 (ICD-10) registered diagnosis of T2D,E11.-
3 with ongoing T2D pharmacological treatment with at least 2 prescriptions in the past 6 months before inclusion.

3 Information from the Swedish National Prescribed Drug Register:
Incident drug prescriptions n=54 insulin, n= 230 Metformin, n=58 Glimepiride, n=1 Metformin + Pioglitazone, n=2 Metformin + Sitagliptin, n=5 Acarbose, n=21 Pioglitazone, n=25 DDP4, n=7 GLP1

© 2024 Zoulakis M et al. *JAMA Network Open.*

eTable 1. Bone Microarchitecture in Older Women with T2D and Controls

Radius ultradistal site	T2D<br>n=238	Control<br>n=2192	p
Cortical Area (mm^2)	40.3 (12.4)	37.2 (11.4)	<.001
Total vBMD (mg/cm^3)	261.1 (65.5)	238.0 (61.7)	<.001
Cortical vBMD (mg/cm^3)	783.1 (79.4)	768.5 (78.7)	<.001
Tr. BV/TV (%)	11.2 (3.4)	9.9 (3.4)	<.001
Tr.Th (mm)	0.592 (0.11)	0.580 (0.11)[b]	.10
Tr.Sp (mm)	0.50 (0.17)	0.58 (0.23)[c]	<.001
Cortical Porosity (%)	4.59 (2.23)[d]	4.43 (2.22)[e]	.30
Stiffness (kN/mm)[a]	59 (14)	54 (12)[b]	<.001
Failure load (N)[a]	3013 (674)	2778 (595)[b]	<.001
Radius distal site	n=270	n=2494	p
Cortical Area (mm^2)	61.6 (9.7)	58.9 (9.6)	<.001
Total vBMD (mg/cm^3)	567.5 (103.9)	528.0 (101.2)	<.001
Cortical vBMD (mg/cm^3)	1009.8 (38.7)	1002.6 (38.7)	.004
Cortical Porosity	2.24 (2.07)[f]	2.39 (1.92)[h]	.26
Stiffness (kN/mm)[a]	71 (11)	68 (11)[i]	<.001
Failure load (N)[a]	3538 (546)	3404 (553)[i]	<.001
Tibia distal site	n=289	n=2669	p
Cortical Area (mm^2)	153.5 (24.0)	147.02 (23.7)	<.001
Total vBMD (mg/cm^3)	410.7 (83.8)	382.0 (77.1)	<.001
Cortical vBMD (mg/cm^3)	925.8 (41.6)	914.4 (41.7)	<.001
Cortical Porosity (%)	4.87 (2.34)[k]	5.38 (2.50)[l]	<.001
Stiffness (kN/mm)[a]	187 (28)	180 (27)[m]	<.001
Failure load (N)[a]	9311 (1390)	8938 (1324)[m]	<.001

Unless otherwise indicated, all values are means (SD). Abbreviations: BV/TV bone volume fraction, HRpQCT= High- resolution peripheral quantitative computed tomography, Tb.Th = trabecular thickness, Tb.Sp = trabecular separation, vBMD = volumetric BMD ultradistal site= standard ultradistal measurement site, distal site = 14% site = measurement at 14% of tibia or radius length. a= Fine element analyses (FEA) , b=2188, c=2180,d=237 ,e=2187, f=268 ,g=2483 , h=2483,i=2492, j=2668, k=287, l=2657, m=266

© 2024 Zoulakis M et al. *JAMA Network Open.*

eTable 2. Fracture Outcomes for Women with T2D vs Controls

	Controls (n=2714)	T2D (n=294)	*p*
**Any fracture**			
Time (years) at risk, median (IQR)	7.3(4.5-8.4)	7.1(3.7-8.1)	
No. (%)	956 (35.2)	115 (39.1)	
Rate (/1000 person years)	55.08	65.48	
HR (95 %)			
Unadjusted	1 (Reference)	1.19 (0.98-1.45)	.07
Adjusted for age, BMI (model 1)	1 (Reference)	1.20 (0.99-1.46)	.07
+ Clinical risk factors (model 2)	1 (Reference)	1.23 (1.01-1.49)	.04
+ FN BMD (model 3)	1 (Reference)	1.26 (1.04-1.54)	.02
**Major osteoporotic fracture**			
Time (years) at risk, median (IQR)	7.5(5.3-8.5)	7.3(4.5-8.2)	
No. (%)	763 (28.1)	90 (30.6)	
Rate (/1000 person years)	41.93	48.27	
HR (95 %)			
Unadjusted	1 (Reference)	1.16 (0.93-1.44)	.19
Adjusted for age, BMI (model 1)	1 (Reference)	1.17 (0.94-1.46)	.17
+ Clinical risk factors (model 2)	1 (Reference)	1.22 (0.97-1.52)	.08
+ FN BMD (model 3)	1 (Reference)	1.25 (1.00-1.56)	.05
**Hip fracture**			
Time (years) at risk, median (IQR)	7.9(7.1-8.8)	7.6(6.8-8.5)	
No. (%)	208 (7.7)	24 (8.2)	
Rate (/1000 person years)	10.13	11.33	
HR (95 %)			
Unadjusted	1 (Reference)	1.15 (0.75-1.75)	.52
Adjusted for age, BMI (model 1)	1 (Reference)	1.28 (0.83-1.97)	.26
+ Clinical risk factors (model 2)	1 (Reference)	1.31 (0.85-2.01)	.22
+ FN BMD (model 3)	1 (Reference)	1.31 (0.86-2.01)	.21

Hazard ratios (HR) and 95% Confidence Interval, Cox proportional hazard models. Unadjusted model, Model 1: adjusted for age and BMI. Model 2: adjusted for age, BMI, and clinical risk factors included in FRAX* and previous treatment with osteoporosis medication. Model 3: all covariates used in model 2 with the addition of femoral neck (FN) BMD, *Previous fracture after 50 years of age, family history of hip fracture, oral glucocorticoid use with at least 5mg daily and for 3 months or more, excessive alcohol intake (21 units per week or more), current smoking, secondary osteoporosis, and rheumatoid arthritis.

© 2024 Zoulakis M et al. *JAMA Network Open.*

eTable 3. Characteristics According to T2D Medication Type

	Insulin N=54	Oral T2D medication N=190	No T2D medication N=50	Controls N=2714	p	Significant differences between groups
Age (years)	77.8 (1.4)	77.8 (1.7)	78.1 (1.8)	77.8 (1.6)	0.66	
Weight (kg)	75.9 (14.2)	75.6 (13.4)	69.8 (12.7)	68.1 (11.8)	**<.001**	Control < Oral, insulin
BMI (kg/m$^{2)}$	29.2 (5.6)	28.8 (4.8)	26.8 (4.5)	26.0 (4.3)	**<.001**	Control < Oral, insulin
BMD FN (g/cm^2)	0.68 (0.12)	0.70 (0.12)	0.64 (0.10)[a]	0.66 (0.10)[b]	**<.001**	Control < Oral, insulin
BMD Total Hip (g/cm^2)	0.84 (0.14)	0.85 (0.13)	0.79 (0.11)[a]	0.80 (0.10)[b]	**<.001**	Control < Oral
BMD Spine (g/cm^2)	1.00 (0.16)[y]	1.01 (0.18)[c]	0.92 (0.17)	0.94 (0.17)[d]	**<.001**	Control < Oral
TBS	1.17 (0.13)[y]	1.20 (0.12)[c]	1.20 (0.11)	1.21 (0.11)[d]	**.01**	Control > insulin
BMSi	77.3 (7.2)[e]	77.5 (9.0)[f]	81.3 (6.1)[g]	78.1 (7.3)[h]	.60	
Tibia bone microarchitecture ultradistal site						
Cortical area (mm^2)	81.5 (26.9)[q]	85.8 (23.7)[w]	77.1 (24.1)	77.6 (23.1)[l]	**<.001**	Control < Oral
Total vBMD (mg/cm^3)	242.8 (57.9)[q]	249.1 (51.0)[w]	223.5 (47.0)	224.0 (47.2)[k]	**<.001**	Control < Oral, insulin
Cortical vBMD (mg/cm^3)	743.8 (72.3)[q]	750.9 (67.4)[w]	738.6 (71.2)[1]	738.3 (68.8)[k]	.11	
Tr.BV/TV (%)	13.3 (3.2)[q]	13.5 (2.9)[w]	11.9 (3.0)	12.1 (2.9)[k]	**<.001**	Control < Oral, insulin
Tr.Th (mm)	0.071 (0.01)[q]	0.071 (0.01)[w]	0.072 (0.01)[1]	0.069 (0.01)[k]	.33	
Tr.Sp (mm)	0.48 (0.13)[q]	0.47 (0.11)[w]	0.56 (0.15)	0.52 (0.13)m	**<.001**	Control > Oral
Cortical porosity (%)	12.3 (3.7)[q]	12.2 (4.1)[w]	12.3 (4.1)[j]	12.2 (3.9)[o]	.99	
Stiffness (kN/mm)	170 (33)[q]	174 (29)[w]	162 (27)	162 (29)	**<.001**	Control < Oral
Failure load (N)	8593 (1590)[q]	8865 (1425[w]	8201 (1290)[j]	8272 (1395)[n]	**<.001**	Control < Oral
Physical activity & performance						
PCS12	37.9 (11.4)	42.1 (11.0)[p]	45.9 (9.8)	45.5 (10.8)[r]	**<.001**	Control > Oral, insulin
PASE, median (IQR)	75.0(50.7-109.3)	85.0(56.4-116.3)	79.0(58.4-131.7)	98.2(67.5-136.0)[s]	**<.001**	Control > Oral, insulin
Timed Up and GO (s)	11.3 (5.7)[y]	9.9 (3.4)	8.4 (2.3)[u]	8.6 (3.1)[v]	**<.001**	Control < Oral, insulin
Walking speed (m/s)	1.08 (0.31)[t]	1.15 (0.24)	1.27 (0.24)[u]	1.27 (0.25)[v]	**<.001**	Control > Oral, insulin
30s Chair stand (n)	8.2 (4.5)	8.8 (4.5)[w]	10.6 (4.3)[u]	10.7 (4.3)[x]	**<.001**	Control < Oral, insulin
Grip strength (kg)	12.3 (5.0)[y]	13.5 (5.3)[z]	14.6 (5.5)[aa]	14.9 (5.5)[o]	**<.001**	Control > Oral, insulin
One Leg standing (s)	8.9 (6.8)[ab]	10.8 (8.0)[ac]	12.3 (9.5)[f]	14.2 (9.7)[ad]	**<.001**	Control > Oral, insulin

Unless otherwise indicated , values are means (SD). Analysis of variance (ANOVA) with Bonferroni post hoc analyses were used to test for significant changes between groups (insulin, oral diabetes treatment, T2D without medication, and non-diabetic controls). Abbreviations: BMD=Bone mineral density, BMSi=Bone material strength index, TBS=Trabecular bone score, HRpQCT= High- resolution peripheral quantitative computed tomography, Tb.N = trabecular number, Tb.Sp = trabecular separation, vBMD = volumetric BMD, BV/TV = bone volume fraction, PCS-12=Physical component score, PASE=Physical Activity Scale for the Elderly.

a= 49, b= 2702, c= 188, d= 2692, e= 12, f= 37, g= 8, h= 587, i= 187, j= 46, k= 2626, l= 2627, m= 2615, n= 2625, o= 2623, p= 189, q= 50, r= 2707, s= 2701, t= 53, u= 49, v= 2695, w= 187, x= 2688, y= 52, z= 178, aa= 47, ab= 27, ac= 125, ad= 2210, ae =2712

© 2024 Zoulakis M et al. *JAMA Network Open.*

eTable 4. Characteristics According to T2D Duration

	Tertile 3<br>N=81	Tertile 2<br>N=82	Tertile 1<br>N=82	Controls<br>N=2714	P	Significant differences between groups
Treatment duration, years (median/IQR)	9.22(1.23)	6.78 (2.32)	2.15(2.33)			
Age (years)	77.8 (1.6)	77.8 (1.7)	77.9 (1.6)	77.8 (1.6)	.96	
Weight (kg)	72.9 (12.9)	77.2 (13.5)	76.6 (13.9)	68.1 (11.8)	**<.001**	0<T1,T2,T3
BMI (kg/m$^{2)}$	28.0 (4.9)	29.7 (4.9)	28.9 (5.0)	26.0 (4.3)	**<.001**	0<T1,T2,T3
BMD Femoral neck (g/cm^2)	0.69 (0.11)	0.70 (0.13)	0.69 (0.11)	0.66 (0.10)[a]	**<.001**	0<T1,T2,T3
BMD Hip total (g/cm^2)	0.84 (0.14)	0.85 (0.13)	0.84 (0.14)	0.80 (0.11)[a]	**<.001**	0<T1,T2,T3
BMD Lumbar spine (g/cm$^{2)}$	1.00 (0.15)[e]	1.01 (0.20)	1.01 (0.19)[f]	0.94 (0.17)[d]	**<.001**	0<T1,T2,T3
TBS	1.20 (0.10[e]	1.18 (0.13)[c]	1.19 (0.12)[f]	1.21 (0.11)[d]	**.04**	
BMSI	77.4 (6.5)[g]	78.5 (9.7)[h]	76.9 (10.0)[i]	78.1 (7.3)[j]	.88	
Tibia bone microarchitecture ultradistal site						
Cortical area (mm^2)	81.70 (24.20)[e]	87.5 (27.3)[e]	85.5 (21.2)[f]	77.6 (23.1)[l]	**<.001**	0<T1,T2
Total vBMD (mg/cm^3)	246.2 (55.5)[e]	248.7 (55.5)[e]	247.3 (47.5)[f]	224.0 (47.2)[k]	**<.001**	0<T1,T2,T3
Cortical vBMD (mg/cm^3)	745.1 (68.0)[e]	749.7 (75.8)[e]	753.3 (60.8)[f]	738.3 (68.8)[k]	.10	
Tr.BV/TV (%)	13.6 (3.2)[e]	13.2 (2.9)[e]	13.5 (2.9)[f]	12.1 (2.9)[k]	**<.01**	0<T1,T2,T3
Tr.Th (mm)	0.073 (0.01)[e]	0.070 (0.01)[e]	0.069 (0.01)[f]	0.069 (0.01)[k]	**.02**	0<T3
Tr.Sp (mm)	0.49 (0.13)[e]	0.47 (0.11)[e]	0.46 (0.12)[f]	0.52 (0.13)[m]	**<.001**	0>T1,T2
Cortical Porosity (%)	12.7 (4.1)[e]	12.4 (4.0)[e]	11.8 (3.9)[f]	12.2 (3.9)[o]	.57	
Stiffness (kN/mm)	172 (33)[e]	173 (29)[e]	175 (29)[f]	162 (29)[n]	**<.001**	0<T1,T2,T3
Failure load (N)	8728 (1580)[e]	8791 (1391)[e]	8900 (1424)[f]	8272 (1395)[n]	**<.001**	0>T1,T2,T3
Physical Activity & Performance						
PCS12	39.7 (10.6)[f]	39.9 (11.1)	44.0 (11.7)	45.5 (10.8[p]	**<.001**	0>T2,T3
PASE, median (IQR)	79.2(55-113.0)[f]	83.0(52.1-123.6)	84.6(58.6-114.6)	98.2(67.5-136.0)[q]	**<.001**	0>T2,T3
Timed Up and Go (s)	10.5 (4.7)[e]	10.4 (3.7)	9.7 (3.5)	8.6 (3.1[y]	**<.001**	0>T1,T2,T3
30s Chair stand (n)	8.7 (4.5)[u]	8.7 (4.1)	8.6 (5.0)[r]	10.7 (4.3)[r]	**<.001**	0>T1,T2,T3
Walking speed (m/s)	1.12 (0.25)[f]	1.11 (0.27)	1.17 (0.25)	1.27 (0.25)[y]	**<.001**	0>T1,T2,T3
One Leg Standing (s)	10.1 (7.2)[z]	9.7 (7.2)[z]	11.8 (9.4)[z]	14.2 (9.7)[s]	**<.001**	0>T2,T3
Grip strength (kg)	12.4 (5.4)[u]	13.1 (5.2)[v]	14.2 (5.0)[w]	14.9 (5.5)[o]	**<.001**	0>T2,T3

Unless otherwise indicated, values are means (SD) or median (IQR). Analysis of variance followed by Bonferroni post hoc analyses were used to test for significant changes between groups (Controls =0 ,T1=First Tertile, T2=Second Tertile, T3=Third Tertile). Abbreviations: BMD=Bone mineral density, BMSi=Bone material strength index, TBS=Trabecular bone score, HRpQCT= High-resolution peripheral quantitative computed tomography, Tb.Th = trabecular thickness, Tb.Sp = trabecular separation, vBMD = volumetric BMD, PCS-12=Physical component score, PASE=Physical Activity Scale for the Elderly.

a = 2702, d = 2692, e = 79, f = 80, g = 20, h = 11, i = 18, j = 587, k = 2626, l = 2627, m = 2615, n = 2625, o = 2623, p = 2707, q = 2701, r = 2688, s = 2210, t = 2712, u = 78, v = 76, w = 77, y = 2695, z=51.

© 2024 Zoulakis M et al. *JAMA Network Open.*

eTable 5. Characteristics According to Incident Fracture

	T2D With Fracture N=115	T2D Without Fracture N=179	Controls With Fracture N=956	Controls Without Fracture N=1758	p	Significant differences between groups
BMD FN (g/cm^2)	0.66 (0.01)	0.70 (0.12)[a]	0.63 (0.01)[b]	0.67 (0.11)[c]	<.001	2>1,3 & 4>3
BMD Total Hip (g/cm^2)	0.80 (0.11)	0.86 (0.13)[a]	0.76 (0.10)[b]	0.81 (0.12)[c]	<.001	2>1,3,4 & 3<1,4
BMD Spine (g/cm^2)	0.98 (0.15)[d]	1.00 (0.20)	0.91 (.0.15)[e]	0.95 (0.17)[f]	<.001	1>3& 2>3,4 & 4>3
TBS	1.17 (0.12)[d]	1.20 (0.11)	1.20 (0.10)[e]	1.22 (0.11)[f]	<.001	1<4, & 3<4
BMSi	78.9 (8.3)[g]	77.4 (8.3)[h]	78.8 (7.1)[i]	78.1 (7.3)[j]	.34	
Tibia bone microarchitecture ultradistal site	N=110	N=173	N=919	N=1708		
Cortical area (mm^2)	76.9 (23.9)	88.0 (23.9)	73.2 (22.4)	79.9 (23.1)	<.001	1<2,4 &2>3,4 & 3<4
Total vBMD (mg/cm^3)	228.5 (48.4)	253 (52.6)	212.7 (45.5)	212.7 (45.5)[l]	<.001	1<2 & 2>3,4 &3<4
Cortical vBMD (mg/cm^3)	730.6 (70.0)	758.5 (66.0)	728.8 (69.1)[k]	743.4 (68.1)	<.001	2>1,3,4 & 3<4
Tr.BV/TV (%)	12.5 (2.7)	13.6 (3.2)	11.5 (2.9)	12.2 (2.9)[l]	<.001	2>1,3,4 &3<1,3,4
Tr.Th (mm)	0.070 (0.01)	0.071 (0.01)	0.067 (0.01)	0.069 (0.01)[l]	<.001	3<1,2,4
Tr.Sp (mm)	0.51 (0.13)	0.47 (0.12)	0.54 (0.14)[m]	0.51 (0.13)[n]	<.001	2<3,4 & 3<4
Cortical porosity (%)	13.1 (4.2)	11.8 (3.8)	12.2 (3.90)	12.2 (3.9)[o]	.08	
Stiffness (kN/mm)	165 (30)	176 (30)	156 (28)[k]	166 (29)[l]	<.001	2>1,3,4 & 3<1,4
Failure load (N)	8412 (1431)	8905 (1437)	7977 (1352)[k]	8432 (1393)[l]	<.001	2>1,3,4 & 3<1,4
Physical activity & performance	N=114	N=179	N=956	N=1758		
PCS12	39.3 (11.1)	43.6 (10.8)	44.5 (11.2)[q]	46.1 (10.9)	<.001	1<2,3,4 &4>2,3
PASE score, median (IQR)	75.4 (52.1-114.1)	85.0 (58.6-126.9)	90.7 (65.0-132.2)[r]	101.4 (70.8-137.4)[s]	<.001	1<3,4 & 4>3,2
Timed Up and GO (s)	10.9 (4.4)[p]	9.3 (3.3)[a]	9.1 (3.4)[t]	8.4 (3.0)[u]	<.001	1>3,4 & 4<3,2
Walking speed (m/s)	1.08 (0.25)	1.20 (0.25)[a]	1.23 (0.26)[v]	1.29 (0.23)[w]	<.001	1<2,3,4 & 4>2,3
30s Chair stand (n)	7.8 (4.7)[p]	9.7 (4.2)[x]	10.1 (4.5)[y]	11.1 (4.1)[z]	<.001	1<2,3,4 & 4>2,3
Grip strength (kg)	12.2 (5.4)[ab]	14.3 (5.1)[ac]	14.2 (5.6)[ad]	15.2 (5.5)[ae]	<.001	1<2,3,4 & 3<4
One Leg standing (s)	9.4 (7.3)[af]	11.5 (8.6)[ag]	12.9 (9.5)[ah]	14.9 (9.7)[ai]	<.001	1<3,4 & 4>2,3

Values are mean, (SD) unless otherwise specified. Analysis of variance (ANOVA) with Bonferroni post hoc analyses were used to investigate differences between groups (T2D with fracture=1, T2D without fracture=2, controls (non-diabetic) with fracture=3, and controls without fracture=4).
Abbreviations: BMD=Bone mineral density, BMSi=Bone material strength index, TBS=Trabecular bone score, HRpQCT= High- resolution peripheral quantitative computed tomography, Tb.Th = trabecular thickness, Tb.Sp = trabecular separation, vBMD = volumetric BMD, BV/TV = bone volume fraction, PCS-12=Physical component score, PASE=Physical Activity Scale for the Elderly.
a=178, b=953, c=1749, d=111, e=941, f=1751, g=22, h=35, i=183, j=404, k=918, l=1707, m=915, n=1700, o=1704, p=113, q=954, r=951, s=1750, t=947, u=1748, v=949, w=1746, x=176, y=948, z=1740, ab=107, ac=170, ad=926, ae=1697, af=65, ag=124, ah=734, ai=1476.

© 2024 Zoulakis M et al. *JAMA Network Open.*

eTable 6. Baseline Characteristics According to Incident Fracture

	T2D With Fracture N=115	T2D Without Fracture N=179	Controls With Fracture N=956	Controls Without Fracture N=1758	p	Significant differences between groups
Age (years)	78.0 (1.6)	77.7 (1.7)	77.9 (1.6)	77.7 (1.6)	<.001	3>4
Weight (kg)	74.8 (13.6)	74.5 (13.6)	67.9 (11.5)	68.7 (12.1)	<.001	1,2>3,4
BMI (kg/m$^{2)}$	28.5 (4.7)	28.6 (5.1)	25.9 (4.1)	26.1 (4.4)	<.001	1,2>3,4
Current smoking, n(%)	10 (8.7)	6 (3.4)	53 (5.5)	87 (4.9)	.21	
Excessive alcohol intake, n(%)	0 (0)	2 (1.1)	7 (0.7)	8 (0.5)	.48	
Parental hip fracture, n(%)	19 (16.5)	31 (17.3)	176 (18.4)	304 (17.6)	.89	
Previous fractures, n(%)	43 (37.4)	53 (29.6)	456 (47.7)	558 (31.7)	<.001	3>2,4
Vertebral fracture on VFA, n(%)	29 (27.1)[a]	40 (23)[b]	308 (33.1)[c]	325 (19.2)[d]	<.001	3>4
Oral glucocorticoid use, n(%)	2 (1.7)	6(3.4)	33 (3.5)	61 (3.5)	.80	
Secondary osteoporosis, n(%)	25 (21.7)	31 (17.3)	161 (16.8)	325 (18.5)	.51	
Previous osteoporosis medication[c], n(%)	7 (6.1) [a]	3 (1.7)	94 (9.8) [f]	108 (6.2) [g]	<.001	3>2
Rheumatoid arthritis, n(%)	7 (6.1)	6 (3.4)	49 (5.1)	55 (3.1)	.41	
Calcium intake, mg/day, median, (IQR)	364 (0- 653)	344 (0- 598)	327 (0- 620)	419 (0- 626)	<.001	4>2,3
Reported falls the last year, n (%)	48 (42.1%)	47 (26.3%)	328 (34.3%)	464 (26.4%)	<.001	1>2,4 & 3>4
FRAX score, mean (SD), %						
MOF with BMD	23.1 (11.1)	19.9 (11.2)	26.0 (12.6)	21.7 (11.2)	<.001	3>2,4
Hip with BMD	10.8 (10.0)	8.8 (10.4)	13.1 (12.2)	10.1 (10.4)	<.001	3>2,4

Values are means (SD) unless otherwise specified.
Analysis of variance (ANOVA) with Bonferroni post hoc analyses was used to investigate differences between groups (T2D with fracture=1, T2D without fracture=2, controls (non-diabetic) with fracture=3, and controls without fracture=4).
a= 107, b=174, c=931, d=1692, e=114, f=955, g=1755

© 2024 Zoulakis M et al. *JAMA Network Open.*

eTable 7. Fracture Outcomes for Women With T2D vs Controls Without Osteoporosis Treatment at Baseline

	Controls (n=2417)	T2D (n=266)	*p*
**Any fracture**			
Time (years) at risk, median (IQR)	7.3 (4.8-8.4)	7.1 (3.9-8.2)	
No. (%)	814 (33.6)	101 (37.8)	
HR (95 %)			
Unadjusted	1 (Reference)	1.16 (0.91-1.46)	.07
Adjusted for age, BMI (model 1)	1 (Reference)	1.22 (0.99-1.51)	.06
+ Clinical risk factors (model 2)	1 (Reference)	1.25 (1.01-1.54)	.04
+ FN BMD (model 3)	1 (Reference)	1.30 (1.05-1.61)	.01
**Major osteoporotic fracture**			
Time (years) at risk, median (IQR)	7.5 (5.6-8.5)	7.3(4.8-8.3)	
No. (%)	655 (27.1)	79 (29.6)	
HR (95 %)			
Unadjusted	1 (Reference)	1.16 (0.91-1.46)	.22
Adjusted for age, BMI (model 1)	1 (Reference)	1.17 (0.92-1.48)	.20
+ Clinical risk factors (model 2)	1 (Reference)	1.22 (0.96-1.54)	.10
+ FN BMD (model 3)	1 (Reference)	1.27 (1.00-1.61)	.05
**Hip fracture**			
Time (years) at risk, median (IQR)	7.9 (7.1-8.8)	7.7 (6.9-8.6)	
No. (%)	185 (7.6)	23 (8.6)	
HR (95 %)			
Unadjusted	1 (Reference)	1.21 (0.78-1.87)	.39
Adjusted for age, BMI (model 1)	1 (Reference)	1.35 (0.87-2.10)	.18
+ Clinical risk factors (model 2)	1 (Reference)	1.37 (0.88-2.12)	.16
+ FN BMD (model 3)	1 (Reference)	1.41 (0.91-2.18)	.13

Hazard ratios (HR) and 95% Confidence Interval, Cox proportional hazard models
Unadjusted model, Model 1: adjusted for age and BMI. Model 2: adjusted for age, BMI, and clinical risk factors included in FRAX* and previous treatment with osteoporosis medication. Model 3: all covariates used in model 2 with the addition of femoral neck (FN) BMD, *Previous fracture after 50 years of age, family history of hip fracture, oral glucocorticoid use with at least 5mg daily and for 3 months or more, excessive alcohol intake (21 units per week or more), current smoking, secondary osteoporosis, and rheumatoid arthritis.

© 2024 Zoulakis M et al. *JAMA Network Open.*

eTable 8. Fracture Outcomes for Women With T2D vs. Controls Without Previous Osteoporosis Treatment

	Controls (n=2512)	T2D (n=284)	*p*
**Any fracture**			
Time (years) at risk, median (IQR)	7.3 (4.5-8.4)	7.1 (3.9-8.1)	
No. (%)	862 (34.3)	108 (38)	
HR (95 %)			
Unadjusted	1 (Reference)	1.19(0.97-1.45)	.09
Adjusted for age, BMI (model 1)	1 (Reference)	1.19(0.97-1.46)	.09
+ Clinical risk factors (model 2)	1 (Reference)	1.20(0.98-1.47)	.07
+ FN BMD (model 3)	1 (Reference)	1.24(1.01-1.52)	.04
**Major osteoporotic fracture**			
Time (years) at risk, median (IQR)	7.5 (5.5-8.5)	7.3 (4.7-8.2)	
No. (%)	680 (27.1)	83 (29.2)	
HR (95 %)			
Unadjusted	1 (Reference)	1.14(0.91-1.43)	.25
Adjusted for age, BMI (model 1)	1 (Reference)	1.14(0.91-1.44)	.26
+ Clinical risk factors (model 2)	1 (Reference)	1.16(0.92-1.46)	.20
+ FN BMD (model 3)	1 (Reference)	1.20(0.95-1.51)	.12
**Hip fracture**			
Time (years) at risk, median (IQR)	7.9 (7.1-8.8)	7.6 (6.8-8.5)	
No. (%)	185 (7.5)	22 (7.7)	
HR (95 %)			
Unadjusted	1 (Reference)	1.12(0.72-1.75)	.61
Adjusted for age, BMI (model 1)	1 (Reference)	1.24(0.79-1.94)	.35
+ Clinical risk factors (model 2)	1 (Reference)	1.26(0.80-1.96)	.32
+ FN BMD (model 3)	1 (Reference)	1.27(0.81-1.99)	.29

Hazard ratios (HR) and 95% Confidence Interval, Cox proportional hazard models
Unadjusted model, Model 1: adjusted for age and BMI. Model 2: adjusted for age, BMI, and clinical risk factors included in FRAX*. Model 3: all covariates used in model 2 with the addition of femoral neck (FN) BMD,
*Previous fracture after 50 years of age, family history of hip fracture, oral glucocorticoid use with at least 5mg daily and for 3 months or more, excessive alcohol intake (21 units per week or more), current smoking, secondary osteoporosis, and rheumatoid arthritis.

eTable 9. Fractures and Mortality per Tertile of HbA_{1c}

		HbA1c Tertiles		
	**Controls**	**1st n=39**	**2nd n=34**	**3rd n=30**
HbA1c[a], median (IQR)	37 (35- 43)[b]	41 (38- 43)	48 (46-50)	57 (54- 60.5)
MOF, n (%)	763 (28.1)	5 (13)	7 (21)	8 (27)
MOF, HR (95% CI)	Reference	0.55 (0.23-1.35)	0.75 (0.53 to 2.43)	1.74 (0.85 to 3.59)
Any fracture, n (%)	956 (35.2)	9 (23)	11 (32)	14 (47)
Any fracture, HR (95% CI)	Reference	0.74 (0.38-1.46)	1.31 (0.71 to 2.41)	2.34 (1.35 to 4.07)
Hip fracture, n (%)	208 (7.7)	2 (5)	1 (3)	4 (13)
Hip fracture, HR (95% CI)	Reference	0.92 (0.22-3.84)	0.70 (0.10-5.17)	4.56 (1.59-13.03)
Death, n (%)	477 (17.6)	3 (8)	4 (12)	6 (20)
Death, HR (95% CI)	Reference	0.55(0.17-1.72)	0.87(0.32-2.36)	1.58 (0.69-3.64)

The distribution of Hba1c and fractures or mortality is presented per tertile of HbA1c. The HR according to tertile of HbA1c (with the 1st tertile as reference) is presented for MOF, any fracture, hip fracture, and death. The Cox models are fully adjusted (age, BMI, CRFs, and FN BMD). Abbreviations: HbA1c=glycated hemoglobin; MOF=major osteoporotic fracture. a= HbA1c units of measurement = (mmol/mol), b=896. FN=Femoral Neck.

© 2024 Zoulakis M et al. *JAMA Network Open.*

eTable 10. Mortality and T2D

Diabetes and Mortality	Mortality n(%)	HR 95% CI	p
Controls	477 (17.6)	Reference 1	
T2D	79 (26.9)	1.54 (1.21-1.97)	<.001
T2D according to medication			
Controls	477 (17.6)	Reference 1	
Insulin	18 (33.3)	2.01 (1.25-3.23)	.03
Oral medication	47 (24.7)	1.40 (1.03-1.91)	.004
No medication	14 (28.0)	1.57 (0.92–2.67)	.10
T2D according to duration (tertiles)			
Controls	477 (17.6)	Reference 1	
T1	21 (25.6)	1.46 (0.94-2.27)	.90
T2	22 (27.2)	1.43 (0.92-2.21)	.11
T3	22 (27.2)	1.75 (1.12-2.71)	.01
Incident fracture			
Controls without fracture	264 (15)	Reference 1	
Controls with fracture	213 (22.3)	1.32 (1.10-1.59)	.03
T2D with fracture	32 (27.8)	1.30 (0.86–1.97)	.21
T2D without fracture	47 (26.3)	1.64 (1.15-2.33)	.006

The number of events is presented according to diabetes groups. The associations between diabetes and mortality are results from fully adjusted Cox proportional hazards models. Adjusted or age, BMI, FRAX clinical factors, previous osteoporosis medication, and femoral neck BMD. Abbreviation: CI = confidence interval, T1= First Tertile, T2 = Second Tertile, T3= Third Tertile.

© 2024 Zoulakis M et al. *JAMA Network Open.*

eTable 11. Fine and Gray Subdistribution Hazard Ratios for T2D Women vs Controls

Events	Adjusted SHR (95% CI)	p
Any Fracture	1.21 (1.00-1.47)	**.05**
Major Osteoporotic Fracture	1.21 (0.98-1.50)	.09
Hip Fracture	1.25 (0.82-1.91)	.30

Adjusted Subdistrubution (SHR) hazard ratios for T2D women vs controls for fractures while considering the competing risk of death calculated using a Fine and Gray model. Multivariable adjustments for age, sex, BMI, femoral neck BMD, previous treatment with osteoporosis medication and clinical risk factors included in FRAX (previous fracture after 50 years of age, family history of hip fracture, oral glucocorticoid use with at least 5mg daily and for 3 months or more, excessive alcohol intake (21 units per weekor more), current smoking, secondary osteoporosis, and rheumatoid arthritis).

© 2024 Zoulakis M et al. *JAMA Network Open.*

eReferences

1. Washburn RA, McAuley E, Katula J, Mihalko SL, Boileau RA. The physical activity scale for the elderly (PASE): evidence for validity. *J Clin Epidemiol*. 1999;52(7):643-651. doi:10.1016/s0895-4356(99)00049-9

2. Ware JE, Kosinski M, Keller SD. A 12-Item Short-Form Health Survey: Construction of Scales and Preliminary Tests of Reliability and Validity. *Med Care*. 1996;34(3):220-233.

3. Larsson BAM, Johansson L, Mellström D, et al. One leg standing time predicts fracture risk in older women independent of clinical risk factors and BMD. *Osteoporos Int*. 2022;33(1):185-194. doi:10.1007/s00198-021-06039-6

4. Chang CJ, Chang YS, Yang SW. Using single leg standing time to predict the fall risk in elderly. In: IEEE; 2013:7456-7458.

5. Vellas BJ, Wayne SJ, Romero L, Baumgartner RN, Rubenstein LZ, Garry PJ. One-leg balance is an important predictor of injurious falls in older persons. *J Am Geriatr Soc*. 1997;45(6):735-738.

6. Giorgetti MM, Harris BA, Jette A. Reliability of clinical balance outcome measures in the elderly. *Physiother Res Int*. 1998;3(4):274-283. doi:10.1002/pri.150

7. Larsson BAM, Johansson L, Johansson H, et al. The timed up and go test predicts fracture risk in older women independently of clinical risk factors and bone mineral density. *Osteoporos Int*. 2021;32(1):75-84. doi:10.1007/s00198-020-05681-w

8. Barnekow-Bergkvist M, Hedberg G, Pettersson U, Lorentzon R. Relationships between physical activity and physical capacity in adolescent females and bone mass in adulthood. *Scand J Med Sci Sports*. 2006;16(6):447-455. doi:10.1111/j.1600-0838.2005.00500.x

9. Podsiadlo D, Richardson S. The Timed "Up & Go": A Test of Basic Functional Mobility for Frail Elderly Persons. *J Am Geriatr Soc*. 1991;39(2):142-148. doi:10.1111/j.1532-5415.1991.tb01616.x

10. Jones CJ, Rikli RE, Beam WC. A 30-s Chair-Stand Test as a Measure of Lower Body Strength in Community-Residing Older Adults. *Res Q Exerc Sport*. 1999;70(2):113-119. doi:10.1080/02701367.1999.10608028

11. BOHANNON RW. Comfortable and maximum walking speed of adults aged 20—79 years: reference values and determinants. *Age Ageing*. 1997;26(1):15-19. doi:10.1093/ageing/26.1.15

12. Watson MJ. Refining the Ten-metre Walking Test for Use with Neurologically Impaired People. *Physiotherapy*. 2002;88(7):386-397. doi:10.1016/S0031-9406(05)61264-3

© 2024 Zoulakis M et al. *JAMA Network Open.*

13. Pires IM, Lopes E, Villasana MV, Garcia NM, Zdravevski E, Ponciano V. A Brief Review on the Sensor Measurement Solutions for the Ten-Meter Walk Test. *Computers*. 2021;10(4). doi:10.3390/computers10040049

14. Martins JC, Aguiar LT, Lara EM, Teixeira-Salmela LF, Faria CDCM. Assessment of grip strength with the modified sphygmomanometer test: association between upper limb global strength and motor function. *Braz J Phys Ther*. 2015;19.

15. Lorentzon M, Nilsson AG, Johansson H, Kanis JA, Mellström D, Sundh D. Extensive undertreatment of osteoporosis in older Swedish women. *Osteoporos Int*. 2019;30(6):1297-1305. doi:10.1007/s00198-019-04872-4

16. Genant HK, Wu CY, van Kuijk C, Nevitt MC. Vertebral fracture assessment using a semiquantitative technique. *J Bone Min Res*. 1993;8(9):1137-1148. doi:10.1002/jbmr.5650080915

17. Johansson L, Johansson H, Axelsson KF, et al. Improved fracture risk prediction by adding VFA-identified vertebral fracture data to BMD by DXA and clinical risk factors used in FRAX. *Osteoporos Int*. 2022;33(8):1725-1738. doi:10.1007/s00198-022-06387-x

18. Johansson L, Sundh D, Magnusson P, et al. Grade 1 Vertebral Fractures Identified by Densitometric Lateral Spine Imaging Predict Incident Major Osteoporotic Fracture Independently of Clinical Risk Factors and Bone Mineral Density in Older Women. *J Bone Miner Res*. 2020;35(10):1942-1951. doi:10.1002/jbmr.4108

19. Nilsson AG, Sundh D, Johansson L, et al. Type 2 Diabetes Mellitus Is Associated With Better Bone Microarchitecture But Lower Bone Material Strength and Poorer Physical Function in Elderly Women: A Population-Based Study. *J Bone Min Res*. 2017;32(5):1062-1071. doi:10.1002/jbmr.3057

20. Jaiswal R, Zoulakis M, Axelsson KF, et al. Increased Bone Material Strength Index Is Positively Associated With the Risk of Incident Osteoporotic Fractures in Older Swedish Women. *J Bone Miner Res*. 2023;38(6):860-868. doi:10.1002/jbmr.4816

21. Pistoia W, van Rietbergen B, Lochmüller EM, Lill CA, Eckstein F, Rüegsegger P. Estimation of distal radius failure load with micro-finite element analysis models based on three-dimensional peripheral quantitative computed tomography images. *Bone*. 2002;30(6):842-848. doi:10.1016/S8756-3282(02)00736-6

© 2024 Zoulakis M et al. *JAMA Network Open.*
